# Image registration for accurate electrode deformation analysis in *operando* microscopy of battery materials

**DOI:** 10.1107/S1600577524012293

**Published:** 2025-02-04

**Authors:** Tianxiao Sun, Robert Peng, Wenlong Li, Yijin Liu

**Affiliations:** a Walker Department of Mechanical Engineering The University of Texas at Austin Austin TX78712 USA; b McNeil High School, Austin, TX78729, USA; Paul Scherrer Institut, Switzerland

**Keywords:** *operando* imaging, battery degradation, image registration, optical flow analysis, chemomechanical coupling

## Abstract

*Operando* imaging techniques have become increasingly valuable in both battery research and manufacturing. However, the reliability of these methods can be compromised by instabilities in the imaging setup and *operando* cells, particularly when utilizing high-resolution imaging systems. The acquired imaging data often include features arising from both undesirable system vibrations and drift, as well as the scientifically relevant deformations occurring in the battery sample during cell operation. For meaningful analysis, it is crucial to distinguish and separately evaluate these two factors. To address these challenges, we employ a suite of advanced image-processing techniques. These include fast Fourier transform analysis in the frequency domain, power spectrum-based assessments for image quality, as well as rigid and non-rigid image-registration methods. These techniques allow us to identify and exclude blurred images, correct for displacements caused by motor vibrations and sample holder drift and, thus, prevent unwanted image artifacts from affecting subsequent analyses and interpretations. Additionally, we apply optical flow analysis to track the dynamic deformation of battery electrode materials during electrochemical cycling. This enables us to observe and quantify the evolving mechanical responses of the electrodes, offering deeper insights into battery degradation. Together, these methods ensure more accurate image analysis and enhance our understanding of the chemomechanical interplay in battery performance and longevity.

## Abstract 2

We applied advanced image-processing techniques, including fast Fourier transform analysis, image registration and optical flow, to mitigate artifacts caused by system instabilities and accurately track battery electrode deformations during operation. This approach improves the reliability of high-resolution *operando* imaging, providing deeper insights into battery degradation and enhancing our understanding of chemomechanical interactions in battery performance.

## 1. Introduction

Batteries are essential to modern energy storage systems, yet their internal behaviors are governed by complex, multi-scale processes, ranging from atomic level ion diffusion to macroscopic mechanical deformation (Kang & Ceder, 2009 ▸; Park *et al.*, 2013 ▸; Jain *et al.*, 2022 ▸). Understanding these processes, which evolve dynamically during battery operation, is crucial for improving performance and extending lifespan (Li *et al.*, 2022 ▸; Dai *et al.*, 2024 ▸; Sun, Sun *et al.*, 2021 ▸). However, traditional post-mortem analysis techniques often fail to capture the real time structural and chemical changes, as they only provide static snapshots of the battery at specific points in time.

To address these limitations, *operando* imaging techniques have become indispensable in battery research, allowing researchers to monitor the evolution of materials during operation (Lim *et al.*, 2016 ▸). These methods provide real time insights into phenomena such as phase transformations (Wang *et al.*, 2016 ▸; Kao *et al.*, 2010 ▸), crack propagation (Yuan *et al.*, 2021 ▸) and electrode deformation (Chen *et al.*, 2014 ▸), all of which are critical for understanding battery degradation mechanisms. Among these techniques, synchrotron-based *operando*X-ray microscopy (XM), particularly transmission X-ray microscopy (TXM), is highly valued for its ability to non-destructively visualize the internal structures of battery materials (Xie *et al.*, 2016 ▸). TXM allows researchers to probe both surface and bulk properties of electrode materials, revealing crucial particle-level transformations and mechanical deformations during charge and discharge cycles (Nelson Weker & Toney, 2015 ▸).

Despite its capabilities, *operando*X-ray imaging poses technical challenges. Achieving high spatial resolution requires precision control over the imaging system, and even minor mechanical instabilities such as motor vibrations or sample misalignment, and can introduce artifacts like image blurring or displacement (Sun, Zhang *et al.*, 2021 ▸). These artifacts degrade image quality, complicate data interpretation and obscure subtle yet critical structural changes in the battery (Fu *et al.*, 2021 ▸). Additionally, miniaturized battery cell design is often used to expose a small sample volume to the X-ray probe to facilitate high-resolution imaging experiments (Zhou & Wang, 2021 ▸). Despite tremendous research and engineering efforts, the stability and electrochemical representativeness of these cells are still non-optimal, leading to various types of image artifacts. Such issues are particularly problematic in dynamic, real time studies where even small errors can propagate through data reconstructions, leading to inaccurate conclusions about material behavior.

To overcome these challenges, advanced image-processing techniques are essential. Image-registration methods can be used to correct for frame-to-frame positional shifts caused by mechanical instability, ensuring that structural changes observed over time are accurate (Zhang *et al.*, 2021 ▸). Additionally, fast Fourier transform (FFT)-based image quality assessment allows for the identification and correction of resolution degradation caused by system vibrations, preventing blurred images from corrupting the reconstruction process (Wang *et al.*, 2012 ▸). Optical flow analysis could enhance the capability to track particle motion and electrode deformation in real time, providing detailed information about how mechanical stresses evolve within the battery during operation (McGuire *et al.*, 2016 ▸; Kale *et al.*, 2015 ▸). This method is crucial for understanding how localized stresses lead to failure mechanisms like particle fracture, delamination or loss of electrical connectivity.

In this study, we apply advanced image-processing techniques to enhance the accuracy and reliability of *operando*X-ray imaging by addressing system instabilities. Our framework integrates Fourier transform-based image quality assessment with rigid and non-rigid image registration to eliminate artifacts and ensure high-quality data quantification. To validate the versatility of these techniques, we conducted a parallel experiment using optical microscopy. Despite its lower resolution, optical microscopy allowed real time observation of particle motion on the graphite anode surface under similar conditions. Over a 1/20 C charging process, we captured 1200 images, applying the same image registration and optical flow analysis used in the X-ray imaging. This confirmed that our image-processing methods are effective across different imaging platforms, accurately tracking structural deformations at various scales. By integrating these techniques with both *operando*X-ray and optical microscopy, we offer a comprehensive approach for improving data fidelity in real time studies, yielding deeper insights into battery degradation mechanisms. This work underscores the value of combining advanced imaging technologies with rigorous data analysis to better understand battery behavior and develop more durable energy storage systems.

## 2. Techniques for artifact correction and structural dynamics analysis

In *operando* imaging, one of the most significant challenges arises from the introduction of image noise or artifacts due to system instabilities during real time data acquisition. Even minimal vibrations or slight misalignments within the experimental setup can introduce artifacts that obscure the accuracy of the results. This is particularly critical in synchrotron-based XM, a technique broadly used for studying the internal structure of materials such as battery electrodes, with TXM serving as the primary example in this study. TXM relies on the transmission of X-rays through a sample and the use of an objective lens, typically a Fresnel zone plate, to capture the transmitted X-rays emerging from the sample and generate high-resolution images. By means of transmission X-ray tomography, collecting these images at different orientations of the sample with respect to the X-ray direction allows for the reconstruction of the three-dimensional volume of the sample and its internal features. The accuracy of these reconstructions, however, is highly sensitive to the stability of the system, and even minor instabilities can lead to significant artifacts that propagate through the data and compromise the quality of the final analysis.

To ensure reliable and accurate volume reconstruction in X-ray transmission tomography, a series of preparatory and corrective steps are implemented throughout the data collection process. The first step typically involves capturing a reference image with the sample in the beam path. This reference serves as a baseline for detecting and correcting sample-related variations in the X-ray transmission. Following this, an incident intensity image is captured without the sample in place, which allows for the correction of non-sample-related variations in the X-ray beam or detector response. While these steps are essential for minimizing initial sources of error, they do not address the challenges introduced by mechanical instabilities that occur during sample movement. For instance, one common issue arises when the motor responsible for moving the sample is not fully stabilized when the sample re-enters the X-ray beam path. If an image is captured before the sample has come to a complete stop, the resulting image can appear blurred or misaligned. This occurs due to residual motion, which is not accounted for in the simple reference and incident intensity correction steps. Another factor worth highlighting is the use of miniaturized battery cells for TXM imaging. Although TXM imaging of coin cells and pouch cells has been demonstrated when thin electrodes with a monolayer of active particles were used, their high aspect ratio makes it less compatible with tomography measurements. Therefore, specially designed capillary cells have been developed to expose a small piece of electrode specimen to the X-ray probe for the measurements (Qian *et al.*, 2022 ▸). The downside of this is that the thin capillary cell configuration often leads to jeopardized mechanical stability, which could result in inconstant image quality due to unpredictable cell vibrations. The impact of such blurring can significantly degrade the resolution of the reconstructed images, especially in dynamic *operando* experiments where continuous, real time tracking of structural changes is required.

**Figure 1:**
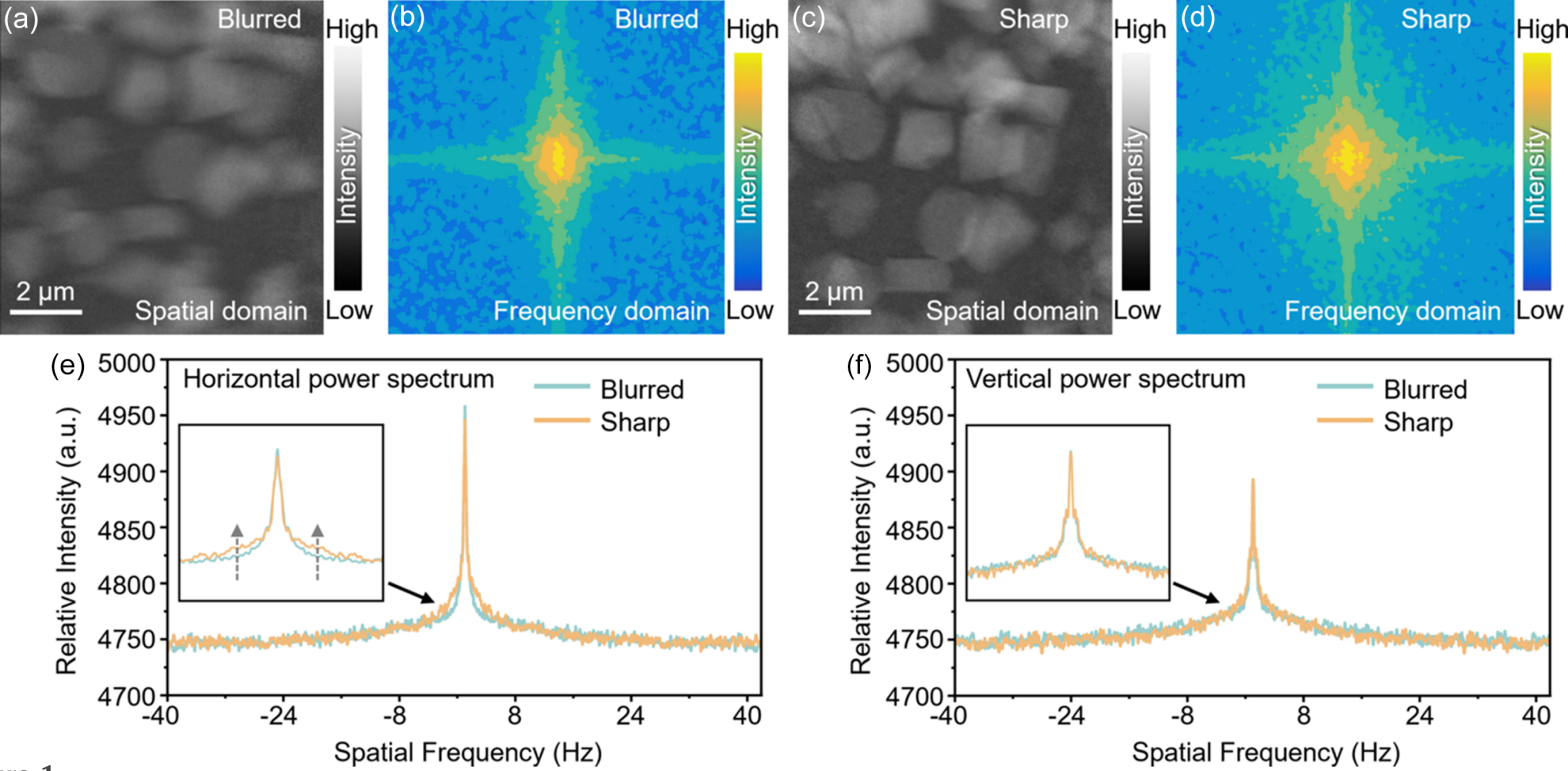
Comparison of blurred and sharp images in real and frequency space due to TXM system instability. (*a*) Blurred real space image caused by system instability in TXM. (*b*) FFT image of the blurred real space image in the frequency domain. (*c*) Sharp real space image without noise. (*d*) FFT image of the sharp real space image in the frequency domain. (*e*) Horizontal power spectrum comparison of the blurred image (*b*) and the sharp image (*d*). The inset shows a magnified view of the region indicated by the solid arrow, highlighting the sharp image’s higher-frequency information in the horizontal direction. The dashed arrow indicates that the sharp image contains more high-frequency information compared with the blurred image within the signal range. (*f*) Vertical power spectrum comparison of the blurred image (*b*) and the sharp image (*d*). The inset shows a magnified view of the region indicated by the solid arrow, demonstrating that there is no significant difference between the sharp and blurred images in the vertical direction.

To investigate the structural evolution of the electrode during the initial charge–discharge cycle, we conducted *operando* TXM experiments at beamline 62C of the Stanford Synchrotron Radiation Lightsource at the SLAC National Accelerator Laboratory. The free-standing cathode sample in an NMC622/Li capillary cell was cycled between 2.8 V and 4.9 V at approximately a 1/12 C rate. X-rays at 8562 eV were selected using a double Si(111)-crystal monochromator to illuminate the sample. Two horizontally adjoining fields of view (FOVs), each 30 µm × 30 µm, were acquired to cover the region of interest. Each image had an exposure time of 48 ms, and we collected images every 15 min with a pixel size of 33.24 nm. To focus on the clearer regions and exclude noisy areas affected by low incident X-ray intensity at the edges of the FOV, we retained only the central region of each image, measuring 654 × 678 pixels, which corresponds to an area of approximately 21.7 µm × 22.5 µm. As shown in Fig. 1 ▸, the effect of system instability is evident in the comparison between blurred and sharp images captured during a TXM experiment. Fig. 1 ▸(*a*) illustrates the degree of blurring introduced by system instability, whereas Fig. 1 ▸(*c*) presents a sharp, well resolved image captured under stable conditions. To quantify and understand the nature of this blurring, we employed a FFT analysis, which allowed us to investigate the frequency domain of the images. The FFT pattern of the blurred image [Fig. 1 ▸(*b*)] reveals a horizontal contraction when compared with the FFT of the sharp image [Fig. 1 ▸(*d*)], indicating an anisotropic reduction in spatial resolution caused by motor jitter. The degree of contraction in the frequency domain directly correlates with the severity of the blurring, providing a quantitative method to assess image quality.

To mitigate these issues, we utilize an FFT power spectrum-based image-screening technique. This method detects specific patterns in the frequency domain that indicate resolution degradation due to mechanical instabilities such as motor vibration or misalignments. As observed in Figs. 1 ▸(*e*) and 1 ▸(*f*), blurred images exhibit distinctive anisotropic features in their Fourier spectra. By automatically identifying and discarding these low-quality images, we can prevent the propagation of errors into the subsequent reconstruction stages. This filtering process ensures that only high-quality, well aligned images contribute to the final reconstruction, thereby improving the overall accuracy and resolution of the analysis. This step is particularly crucial in the study of battery materials, where small structural changes during charging and discharging cycles must be accurately captured to understand degradation mechanisms. Although it is not implemented here, we would like to point out that a deconvolution approach could potentially be used to correct the blurred images. Since the jitter and vibration are not universal throughout all the images, the above-described method can still be utilized to separate the images into a few categories, each with different point-spread function.

**Figure 2:**
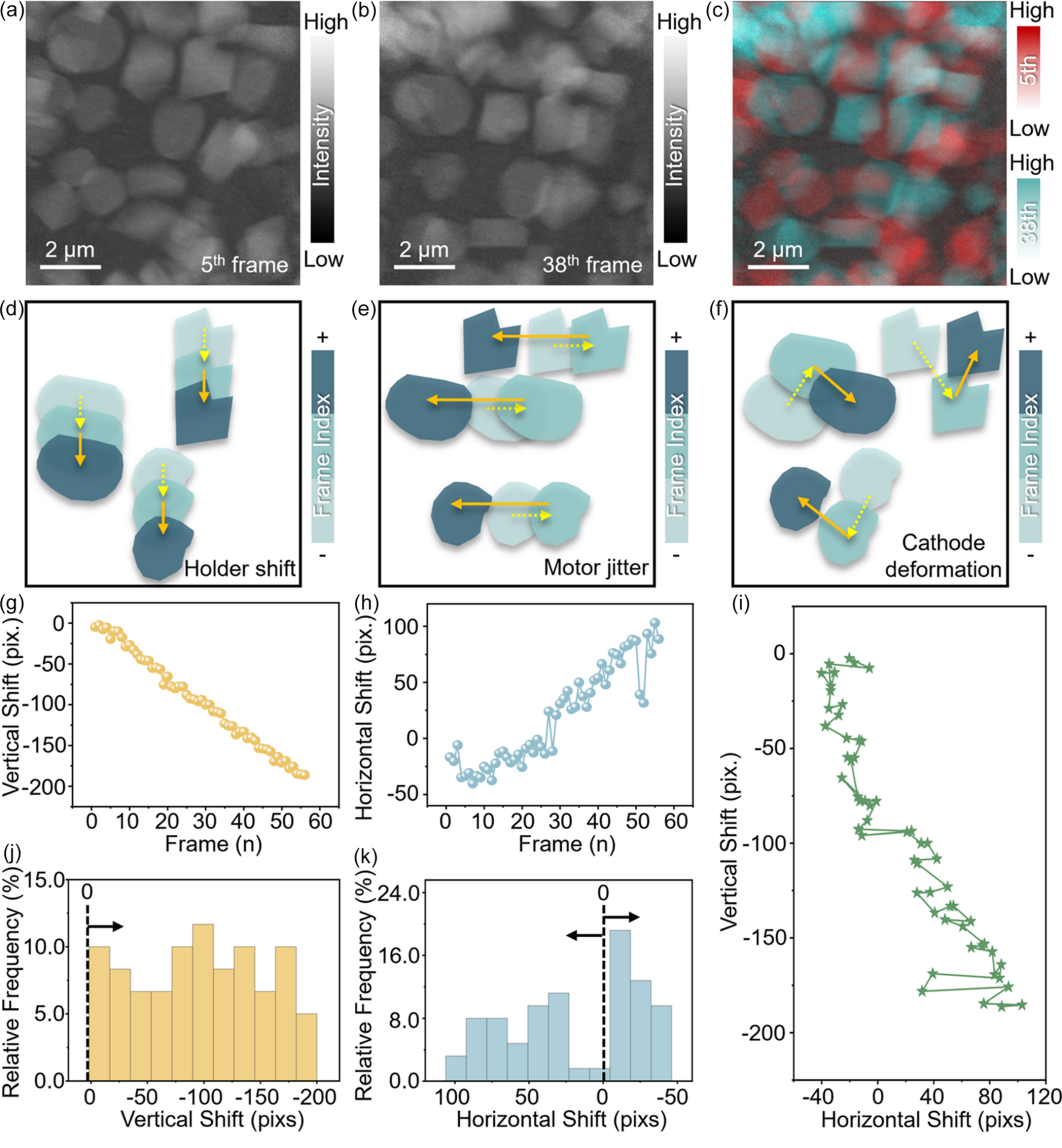
Analysis of positional shifts and deformations in TXM image stacks. (*a*) Image from the 5th frame of the TXM stack. (*b*) Image from the 38th frame of the TXM stack. (*c*) Overlap of the 5th (red) and 38th (blue) frames showing positional differences. (*d*)–(*f*) Schematics of the effects of holder shift, motor jitter and cathode deformation on image displacement. (*g*) Plot of vertical displacements across the stack. (*h*) Plot of horizontal displacements across the stack. (*i*) Displacement path of the images over time. (*j*) Histogram of vertical displacement values. (*k*) Histogram of horizontal displacement values.

In addition to blurring, motor vibrations and sample holder drift can lead to more complex types of image artifacts, such as frame-to-frame displacements, commonly referred to as image jittering and shifting. These displacements introduce positional errors between consecutive frames in a TXM image stack, which could accumulate, complicating the analysis and reconstruction processes. As illustrated in Fig. 2 ▸, such shifts are apparent when comparing frames 5 and 38 of a TXM operando imaging dataset on our capillary battery cell [Figs. 2 ▸(*a*) and 2 ▸(*b*)], with the overlap of these frames [Fig. 2 ▸(*c*)] showing significant displacement. These displacements arise from multiple coupled effects, including sample holder shifts [Fig. 2 ▸(*d*)], motor jitter [Fig. 2 ▸(*e*)] and, in some cases, battery electrode deformation [Fig. 2 ▸(*f*)], such as cathode bending or expansion during electrochemical cycling. For meaningful interpretation, it is important to distinguish and separately analyze these two factors.

To address these positional shifts, we applied a multi-step image-registration process, compensating for both the collective movement of particles within the entire FOV and the localized, scientifically relevant movements across the image sequence. The first step in this process involved using a rigid transformation method to align the images based on collective shifts, primarily attributed to sample holder drift. In the vertical direction [Fig. 2 ▸(*g*)] the images exhibited a continuous drift in the negative direction, with a total displacement of 5.2 µm (187 pixels) over 840 min (56 frames), likely caused by gravitational effects on the cell. In the horizontal direction [Fig. 2 ▸(*h*)] there was a cumulative displacement of 3.0 µm (89 pixels) alongside significant jitter, attributed to the limited precision of the motor as the sample was repeatedly repositioned horizontally to capture images with and without the sample in the beam. The combined sample motion, illustrated in Fig. 2 ▸(*i*), quantitatively reflects the coexistence of systematic drift and random jitter.

This initial rigid registration step effectively corrected for these collective displacements, enabling more precise tracking of localized, scientifically relevant particle movements within the battery electrode. This refinement allowed subsequent analyses to focus on material behavior rather than the stability of the experimental setup.

**Figure 3:**
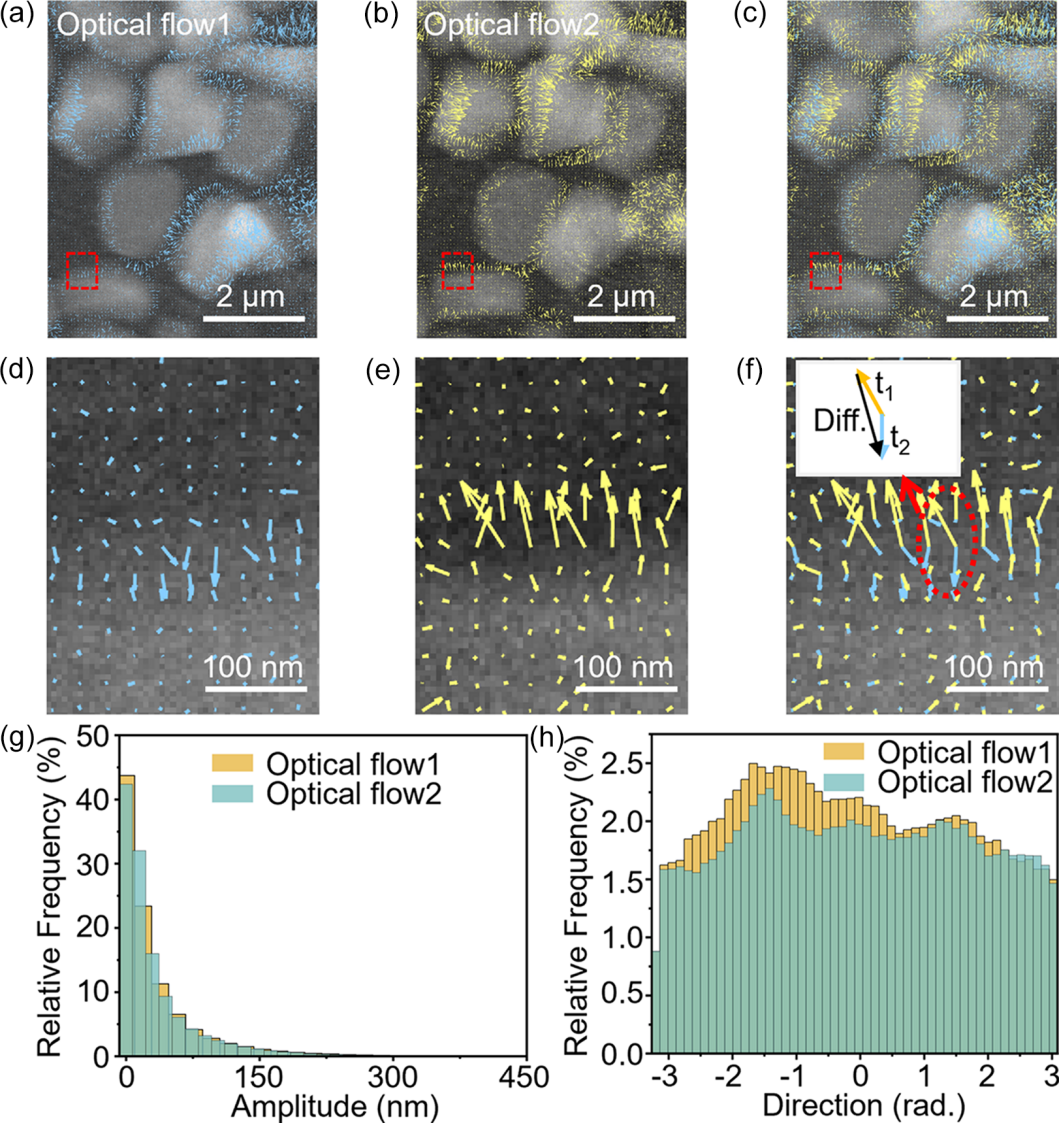
Optical flow analysis of particle motion in TXM image sequence. (*a*) Optical flow field between frames 3 and 13. (*b*) Optical flow field between frames 13 and 35. (*c*) Overlap of the two optical flow fields from (*a*) and (*b*). (*d*)–(*f*) Enlarged views of the R1 regions from (*a*), (*b*) and (*c*), respectively; (*g*)–(*i*) Enlarged views of the R2 regions from (*a*), (*b*) and (*c*), respectively. (*j*) Histogram of the amplitude distribution for the two optical flow fields. (*k*) Histogram of the directional distribution for the two optical flow fields.

Once the rigid image-registration process was complete, we shifted our attention to tracking the dynamic movement of the battery electrode materials using optical flow analysis. This technique quantifies the motion of particles between consecutive images, providing valuable insights into how electrode materials deform in response to the stresses induced by charge and discharge cycles. Fig. 3 ▸ highlights the results of optical flow analysis, with Figs. 3 ▸(*a*) and 3 ▸(*b*) showing the calculated flow fields between frames 3 and 13, and frames 13 and 35, respectively. The overlap of these fields [Fig. 3 ▸(*c*)] illustrates the evolution of particle movement over time, revealing patterns of deformation within the electrode material. Zoomed-in regions of the optical flow fields [Figs. 3 ▸(*d*)–3 ▸(*i*)] provide detailed insights into localized particle motion, whereas amplitude and directional histograms [Figs. 3 ▸(*g*) and 3 ▸(*h*)] quantify the probability distributions of the magnitude and direction of these movements. This analysis revealed heterogeneous deformation patterns, indicating that different regions of the electrode experience varying degrees of mechanical stress during operation. Such information is critical for understanding the mechanisms that drive battery degradation and failure, as localized stresses often lead to mechanical fractures or delamination within the electrode, directly impacting battery performance and longevity.

To complement the TXM analysis, we conducted an in-depth study of the graphite anode electrode’s structural dynamics using optical microscopy. The imaging was performed in reflection mode with an Olympus BX51 microscope (Fig. S1 of the supporting information), equipped with a 10× eyepiece and an Olympus UMPlanFl 10× objective lens (numerical aperture, NA = 0.3). The theoretical resolution of the objective, calculated using Abbe’s diffraction limit (*d* = 0.61λ/NA, where λ = 550 nm for visible light), is approximately 1.1 µm. The FOV of the objective, considering a 10× magnification and standard eyepiece, spans approximately 2 mm × 2 mm. The recorded images had a pixel size of 0.56 µm, allowing for detailed analysis of the particle motion. From these images, we selected a region of interest measuring 180 × 300 pixels (100 µm × 166.7 µm) for further study.

**Figure 4:**
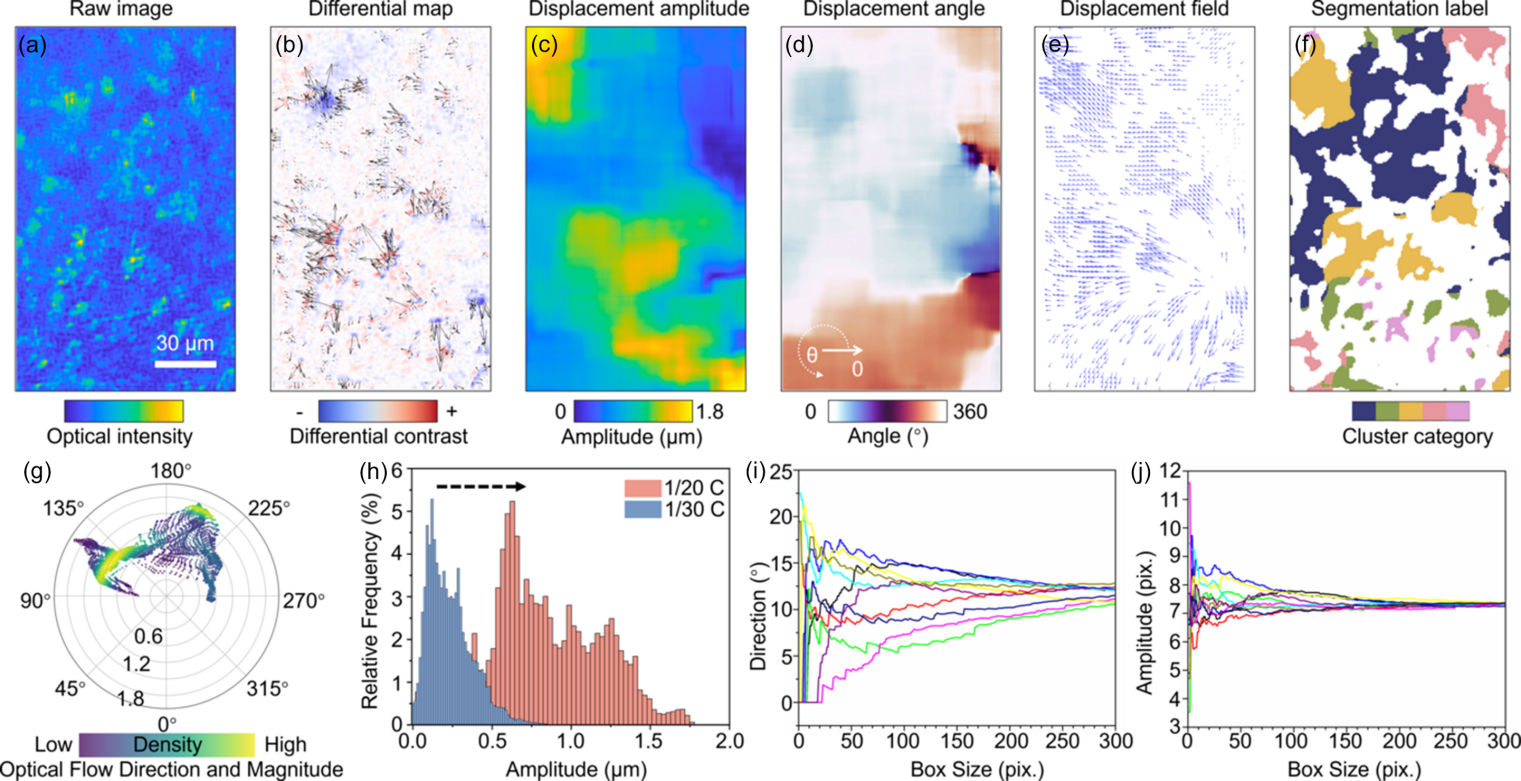
Particle motion analysis in graphite anode during charge and discharge cycles. (*a*) Morphology of graphite anode particles captured by an optical microscope. (*b*) Differential map showing particle motion before and after movement. (*c*) Amplitude distribution of the differential map. (*d*) Directional distribution of the differential map. (*e*) Optical flow matrix map representing particle motion. (*f*) Segmentation of the optical flow matrix, with different colors representing distinct clusters. (*g*) Compass plot illustrating the direction and magnitude of the optical flow. (*h*) Histogram of particle motion amplitudes observed during electrochemical cycling at 1/20 C and 1/30 C rates. (*i*)–(*j*) Plots showing the trend of the (*i*) average optical flow direction and (*j*) amplitude as a function of box size in defined regions.

This method enabled real time imaging of particle motion during electrochemical cycling, with 1200 images captured over a 1/20 C charging period at a rate of one image per minute. After applying the same image registration and optical flow techniques, we successfully tracked the motion of individual particles within the anode, revealing highly heterogeneous motion patterns. Fig. 4 ▸ illustrates the results of this analysis, providing insights into the motion behaviors of the anode electrode during cycling. Fig. 4 ▸(*a*) shows the raw image of the electrode before cycling, serving as the baseline for subsequent comparisons. Fig. 4 ▸(*b*) presents a differential map obtained by subtracting the pre-cycling raw image from the post-charging image; warm colors represent regions where particles appeared (positive values) and cool colors indicate areas where particles disappeared (negative values). The black arrows overlaying Fig. 4 ▸(*b*) depict the displacement vectors, illustrating the direction and magnitude of particle motion between the regions of disappearance and appearance. Fig. 4 ▸(*c*) provides a mapping of displacement magnitudes calculated using the optical flow method, and Fig. 4 ▸(*d*) displays the distribution of displacement angles derived from the same analysis. Fig. 4 ▸(*e*) shows the optical flow field within the particle regions, using a mask generated from the particle area in Fig. 4 ▸(*a*). Here, the arrow lengths correspond to displacement magnitudes, and their orientations indicate displacement directions. Through clustering analysis, the particle regions are categorized into five distinct clusters, which are visually differentiated by colors in Fig. 4 ▸(*f*). Additionally, Fig. 4 ▸(*g*) uses a polar plot to quantitatively display the optical flow field, highlighting the direction and magnitude of particle motion. The analysis reveals that many of the irregularly shaped regions showed coherent particle movement, indicating the structural stiffness within these regions. The correlation among them, on the other hand, can be independent or even in opposing motions. A good analogy of this phenomenon is the tectonic plate shifts, which leads to a buildup of mechanical stress and its release through mechanical fracturing.

To validate this observation, we also conducted cycling experiments at a 1/30 C rate and observed similar particle motion behaviors (Fig. S2 of the supporting information). Under the lower current rate, the distribution of particle motion amplitudes exhibited the same trend as seen at 1/20 C, although the amplitudes were confined to a narrower range [Fig. 4 ▸(*h*)]. This indicates that the observed heterogeneity in particle motion is intrinsic to the structural dynamics of the electrode, rather than being solely dependent on the cycling rate. These findings suggest that the deformation of the anode occurs in stages, with certain regions undergoing more severe mechanical stress than others. This complexity highlights the need for multi-scale analysis in battery research, as localized deformation can have significant implications for overall battery performance.

To further quantify these electrode deformation behaviors, we analyzed the amplitude and angle of particle motion within ten randomly selected regions, and we increase the sampling size progressively [Figs. 4 ▸(*i*) and 4 ▸(*j*)]. Our analysis revealed that, as the sampling size increased, the deviation in deformation measurements decreased, suggesting the existence of a minimum representative volume required to capture the full extent of electrode deformation in the studied system. This insight is critical for designing more reliable models of battery performance and degradation, as it emphasizes the importance of capturing localized mechanical behavior over a sufficiently large sampling volume to ensure accurate predictions of long-term battery performance.

## 3. Conclusions

In this study, we demonstrated the use of advanced imaging and image-processing techniques to overcome the technical challenges associated with *operando* microscopic investigation of battery materials. By implementing Fourier transform-based image screening and image-registration techniques, we were able to screen and control the quality of TXM data, ensuring that only scientifically relevant information was analyzed. We further utilized a rigid image-registration approach to distinguish and separate the effects of setup instability and drift from the structural deformation of battery electrode materials. The latter is relevant to the battery degradation and is subjected to further analysis.

The application of optical flow analysis provided detailed insights into the heterogeneous motion of particles within the electrode, revealing complex mechanical interactions that occur during battery operation. This ability to observe and quantify electrode deformation in real time is critical for understanding the mechanisms of battery degradation and for developing more robust, longer-lasting energy storage systems.

The techniques presented here can be further improved by incorporating more advanced computer vision methods such as deep-learning-based image registration and analysis. As battery research continues to evolve, the integration of advanced imaging technologies and data-processing methods will be essential for addressing the challenges of understanding multi-scale dynamics in complex energy storage materials.

## Supplementary Material

Supporting Figures S1 and S2. DOI: 10.1107/S1600577524012293/gy5070sup1.pdf
