# Supplementary material for: Image registration for accurate electrode deformation analysis in operando microscopy of battery materials
Source: J Synchrotron Radiat. 2025 Feb 4;32(Pt 2):417–23. doi: 10.1107/S1600577524012293 (PMC11892904; doi:10.1107/S1600577524012293)
Supplement: Supplementary file 1 [file s-32-00417-sup1.pdf]

JOURNAL OF SYNCHROTRON RADIATION

Volume 32 (2025)

Supporting information for article:

Image Registration for Accurate Electrode Deformation Analysis in *Operando* Microscopy of Battery Materials

Tianxiao Sun, Robert Peng, Wenlong Li and Yijin Liu

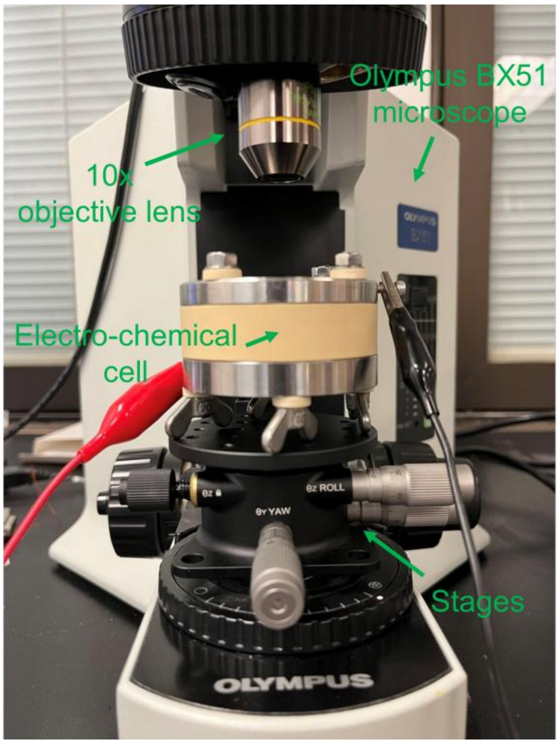

Figure S1 Optical microscope setup for in situ observation. The custom-designed electrochemical cell operates in reflection mode during cycling, utilizing an Olympus BX51 microscope equipped with a 10× eyepiece and an Olympus UMPlanFl 10× objective lens.

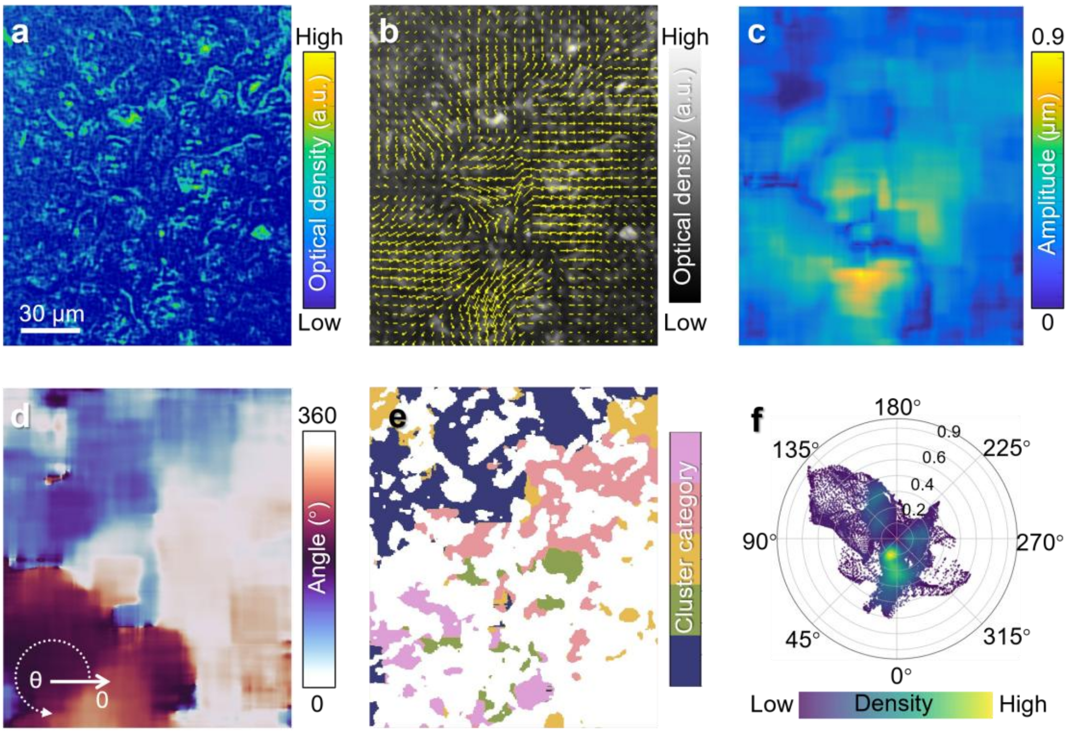

Figure S2 Optical microscopy experiments at a 1/30 C rate. (a) Raw image of the electrode before cycling, serving as the baseline for subsequent comparisons. (b) Optical flow field showing particle motion before and after cycling, where arrow lengths represent the magnitude of motion, and arrow directions indicate motion directions. (c) Mapping of the optical flow magnitude across the electrode. (d) Distribution of motion directions extracted from the optical flow field. (e) Cluster analysis of particle regions based on displacement magnitude and direction. (f) Quantitative analysis of particle motion direction and magnitude in polar coordinates.
